# Development and Optimization of *Andrographis paniculata* Extract-Loaded Self-Microemulsifying Drug Delivery System Using Experimental Design Model

**DOI:** 10.3390/pharmaceutics16020166

**Published:** 2024-01-24

**Authors:** Chaiyakarn Pornpitchanarong, Prasert Akkaramongkolporn, Nattawat Nattapulwat, Praneet Opanasopit, Prasopchai Patrojanasophon

**Affiliations:** Pharmaceutical Development of Green Innovations Group (PDGIG), Faculty of Pharmacy, Silpakorn University, Nakhon Pathom 73000, Thailand; pornpitchanaron_c@su.ac.th (C.P.); akkaramongkol_p@su.ac.th (P.A.); nattapulwat_n@su.ac.th (N.N.); opanasopit_p@su.ac.th (P.O.)

**Keywords:** *Andrographis paniculata*, andrographolide, self-microemulsifying drug delivery system, experimental design

## Abstract

The objectives of this study were to develop an optimized formulation for an *Andrographis paniculata* extract (AGPE)-loaded self-microemulsifying drug delivery system (SMEDDS) using an experimental design and evaluate the characteristics of the developed SMEDDS. The solubility of andrographolide (AGP) in various solvents was investigated. The pseudo-ternary phase was constructed to provide an optimal range for each component to form microemulsions (MEs). The formulation was optimized using an I-optimal design mixture type, where the physical stability, droplet size, polydispersity index, and zeta potential were examined. Soft capsules of the optimized AGPE-loaded SMEDDS were manufactured. The dissolution and ex vivo membrane permeation were studied. Oleic acid, Tween^®^ 80, and PEG 400 were the best solubilizers for AGP. The promising surfactant to co-surfactant ratio to generate ME was 3:1. The optimized SMEDDS contained 68.998% Tween^®^ 80, with 13.257% oleic acid and 17.745% PEG 400. The assayed content of AGP, uniformity of dosage unit, and stability complied with the expected specifications. The dissolution and membrane permeability of AGPE-loaded SMEDDS was significantly improved from the *A. paniculata* extract (*p* < 0.05). All in all, the developed optimized AGPE-loaded SMEDDS was proven to contain optimal composition and AGP content where a stable ME could spontaneously be formed with enhanced delivery efficacy.

## 1. Introduction

*Andrographis paniculata* (Burm. F.) Wall ex Nees is an herbaceous plant in the Acanthaceae family. In China, India, Thailand, Malaysia, and other countries in the area, the plant has long been used to treat upper respiratory infections, flu, sore throats, cold sores, and fever [1]. Apart from that, *A. paniculata* is known to possess several other biological benefits such as anti-inflammatory, antioxidant, anticancer, antiviral, antimicrobial, antihyperglycemic, hepatoprotective, immunostimulatory effects, etc. [2,3]. The phytochemicals contained in *A. paniculata* and its extracts are composed of diterpenoids, diterpene glycosides, lactones, flavonoids, and flavonoid glycosides [4]. The pharmacological activity of *A. paniculata* is mostly attributed to andrographolide (AGP), which is a diterpenoid found especially in the leaves of the plant. Most recently, with the outbreak of coronavirus 2019 (COVID-19, SARS-CoV-2), AGP was found to be effective against the pandemic virus, and several authorities allowed its use with the plant crude extracts to cope with the situation [5]. Research from Sa-ngiamsuntorn et al., (2021) showed that AGP has a potent anti-SARS-CoV-2 activity with an IC_50_ equivalent to the antiviral agent remdesivir [6]. *A. paniculata* also showed clinical efficacy, safety, and improved overall health in the acute phase of mild COVID-19 [7].

AGP has been proven to possess tremendous pharmacological benefits; however, the efficacy of the compound cannot be maximized in terms of drug delivery. Despite the origin of *Andrographis paniculata* and genetic variations which may affect the amount of AGP found in the plant, the compound is poorly soluble in water, but can be dissolved in various organic solvents, e.g., acetone, chloroform, ether, and hot ethanol [1,8,9]. Also, the content of AGP by weight of *A. paniculata*, though varied in different parts of the plant, is very low. The highest amount of AGP that can possibly be found is 6% of the dried leaves weight and even lower in the stem (0.8–1.2%), while only 4% is contained in the whole plant [10]. Moreover, the recommended daily dose of *A. paniculata* crude or even the plant extract would require multiple tablets or capsules per dose to achieve an effective treatment (varied according to illness) due to the low amount of active compound by its weight and low bioavailability of AGP [7,11,12]. Commercially available dosage forms of *A. paniculata* crude or extract are mainly composed of solid dosage forms such as capsules and tablets; liquid dosage forms as in syrups, tinctures, and injectables; or semisolid dosage forms, e.g., creams and ointments [13]. Therefore, the development of a novel suitable formulation that would increase the solubility of AGP and improve the bioavailability of the compound is vital.

Due to the advantageous properties of AGP, many researchers have made attempts to improve the content of AGP in *A. paniculata* and its extracts. For example, exogenous synthetic inducers have been given to the plant to induce leaf stress and chlorophyll reduction and enhance the AGP content per plant dry weight [14]. Novel drug delivery systems have also been studied in various forms to improve AGP solubility and encourage drug delivery effectiveness. Particularly, nano-based systems have been studied and reported, for example, polymeric nanoparticles [15], micelles [16], lipid-based nanoparticles [17,18], inorganic/metallic nanoparticles [19,20], nanoencapsulations [21], nanoemulsions [22], self-nanoemulsifying drug delivery systems [23], and hydrogels [24]. Moreover, AGP derivatives have been developed to increase bioavailability and druggability for various therapeutic purposes [25,26]. Although the development of advanced systems and technologies has shown to be significantly beneficial and promising, further pharmacokinetic and pharmacological examinations of these novel nano-based systems are crucial prior to clinical trials. Furthermore, the pharmaceutical industry today lacks proper methods and instruments for scaling up operations of nanotechnologies, and the cost involved in product development is yet to be discovered [13].

Self-microemulsiying drug delivery system (SMEDDS) refers to homogeneous mixtures of natural or synthetic oils with surfactants and co-surfactants that could easily form a microemulsion (ME) upon mild agitation in the gastrointestinal fluid. Droplets of SMEDDS are considered small particles or droplets, with an actual size of less than 250 nm, with a thermodynamically stable colloidal dispersion system [27]. The ME formed enormously increases the surface area from which the partition of the drug from the oil phase eases into the aqueous phase [28]. The system is suitable for poorly water-soluble drugs where the dissolution limits the drug absorption [29]. The use of SMEDDS bypasses the dissolution obstacle and allows the drug to be absorbed after oral administration. Accordingly, the system improves the oral bioavailability of a compound and enhances the pharmacokinetic profile of the drug [30,31]. Generally, pseudo-ternary phase diagrams are generated to determine the area, stating the amount of oil, surfactant mixture, and water that gives a stable ME. The compositions are then usually non-specially picked from the area, whereas the optimal or most suitable ME components can actually be determined by an experimental design.

In this work, AGPE-loaded SMEDDS has been developed from an optimized oil and surfactant composition obtained using an experimental design. The solubility of AGP from *A. paniculata* extract in several oils, surfactants, and co-surfactants was investigated. Pseudo-ternary phase diagrams for ME formulation were constructed, and the optimized SMEDDS composition was obtained from an I-optimal design mixture type. Also, the formulation was manufactured as soft capsules in an industrial pilot scale, with the assay, uniformity of dosage unit, and stability investigated. To the best of our knowledge, the AGPE formulation with proposed optimization and its scale-up demonstration has not yet been reported.

## 2. Materials and Method

### 2.1. Materials

Andrographolide (AGP) standard and oleic acid were purchased from Sigma Chemical Co. (St. Louis, MO, USA). *A. paniculata* extract (%AGP = 35.84%) was received from Kaewmungkorn Co., Ltd., (Photharam, Ratchaburi, Thailand). Gelatin (bovine hide) for soft capsule production was from Halamix International (Bangkok, Thailand). Tween^®^ 20, Tween^®^ 80, polyethylene glycol (PEG) 400, propylene glycol, and glycerin were from P.C. Drug Center (Bangkok, Thailand). Acetonitrile, ortho-phosphoric acid, ethanol, and methanol were acquired from Merck & Co. (Darmstadt, Germany). Oils were used as commercial food grades, and other chemicals were used as purchased.

### 2.2. Methods

#### 2.2.1. Andrographolide Solubility Study

The solubility of AGP from the dry plant extract in different oils, surfactants, and co-surfactants (as listed in Table 1) was determined. These solvents were selected due to being known as edible, since the formulation was intended to be given orally. An excess amount of the extract was added to 1 mL of each solvent and shaken in an incubator shaker at 37 °C 120 rpm for 72 h. The saturated solutions were collected after centrifugation at 14,000 rpm for 30 min. The obtained supernatants were diluted at least 20× in methanol prior to the quantification of AGP using high-performance liquid chromatography (HPLC).

To find the solubility of AGP in the optimized SMEDDS (highest amount of AGP that can be loaded into the optimized SMEDDS), an excess amount of *A. paniculata* extract was added to the 1.5 mL of the optimized SMEDDS and shaken at 37 °C 120 rpm for 72 h in an incubator shaker. Then, the AGPE-loaded optimized SMEDDS was centrifuged at 14,000 rpm for 30 min to separate the extract powder from the AGP-extracted SMEDDS. The supernatant was collected and diluted with methanol before the analysis of AGP content using HPLC.

#### 2.2.2. HPLC Condition

HPLC analysis with UV detection was used to quantify the content of AGP. An Agilent^®^ 1200 (Santa Clara, CA, USA) with an Agilent^®^ ZORBAX Eclipse XDB-C18 (4.6 × 150 mm, 5 µm) column was used. The mobile phase was methanol:water (65:35), and the injection volume was 20 µL. The flow rate was set at 0.8 mL/min, and the detection wavelength was 228 nm. The standard curve of AGP was constructed, ranging from 5 to 200 µg/mL.

#### 2.2.3. Construction of Ternary Phase Diagram

The ternary phase diagram was constructed to determine the ratio between the surfactant mixtures (S_mix_), oil, and water that could form the stable ME. The S_mix_ was prepared by mixing the surfactant and co-surfactant that provided the highest AGP solubility at certain weight ratios of 1:1, 1:2, 2:1, and 3:1. The ratios were varied according to the preliminarily studies and the results of the solubility studies that could create the ME area under these conditions. Also, they were varied in order to investigate the role of surfactants and co-surfactants in forming a stable ME. The S_mix_ was then mixed with the oil to yield mixtures with different percentages by weight of oil from 10 to 90%. Water was then slowly titrated into each mixture and slowly stirred with a magnetic stirrer. The formation of the ME was visually observed. Accordingly, the transparent colloidal dispersions were considered as MEs, while the turbid solutions formed were accounted as non-microemulsion mixtures. So, the volume of water in which the mixture turned turbid upon the aqueous titration was recorded. The final percentages of water, oil, and S_mix_ in the emulsion were calculated and plotted into the ternary phase diagram to construct a boundary between the ME (transparent) and non-microemulsion (turbid) region using Ternaryplot.com (Jules Blom^©^ 2023). The identified ME area in the pseudo-ternary phase diagram was used as information to find the appropriate range of each component for the experimental design of SMEDDS.

#### 2.2.4. Experimental Design

To formulate the SMEDDS formulation, the volume of water needed to be excess, thus it was not included in the experimental design. Therefore, the mixture components for SMEDDS optimization were surfactant, oil, and co-surfactant that presented the highest AGP solubility, which were oleic acid, Tween^®^ 80, and PEG 400, respectively. In this study, the independent variables for the optimization were X_1_: Tween^®^ 80 (50–80 %wt), X_2_: oleic acid (5–20 %wt), and X_3_: PEG 400 (5–45 %wt). These ranges were selected from the ratios of Smix and the ternary phase diagram, which proved that by mixing these components using each composition within these ranges could form the ME. The dependent variables or the measuring outcomes from the combinations were droplet size Y_1_, polydispersity index (PDI) Y_2_, and zeta potential Y_3_. These parameters were given to Design-Expert^®^ version 11 software, where I-optimal design was used to maintain the total composition of the SMEDDS, generated constant at 100%. A total of 16 runs of varied independent variable compositions were generated. The mixtures of oil, surfactant, and co-surfactant were prepared. An aliquot of each mixture was diluted 1000× with water to confirm the formation of SMEDDS, where a clear transparent solution must be observed prior to further characterization.

#### 2.2.5. Characterizations of SMEDDS

##### Droplet Size, PDI, and Zeta Potential

The dependent variables for SMEDDS optimizations were droplet size, PDI, and zeta potential. These parameters of the generated runs, the optimized SMEDDS, and AGPE-loaded optimized SMEDDS were measured using a light scattering technique by a zetasizer Nano ZS (Malvern Instruments, Malvern, UK). The measurements were conducted in a 1 mL zeta cell at 25 °C and performed in triplicate. The AGPE-loaded optimized SMEDDS were centrifuged at 14,000 rpm for 30 min to remove the excess extract powder prior to the investigation.

##### Physical Stability of the SMEDDS

The physical stability of each prepared SMEDDS was conducted to confirm the formation of ME by the centrifugation method. Briefly, the formulated SMEDDS were transferred into a 1.5 mL microcentrifuge tube and centrifuged at 25 °C and 14,000 rpm for 30 min. Then, the preparations were taken out to observe for any sign of turbidity, creaming, or phase separation.

#### 2.2.6. Formulation of AGP-SMEDDS Soft Capsules

The AGP-SMEDDS soft capsules were manufactured in a pilot-scaled 0.7 kg batch. Roughly, the components of the optimized SMEDDS were weighed and thoroughly mixed, then *A. paniculata* extract was added to obtain a mixture to yield an AGP target content of 30 mg/mL. The extract was shaken for 72 h in an incubator shaker at 120 rpm, and the mixture was centrifuged in an industrial-scale centrifuge at a speed of 14,000 rpm, which was sufficient to remove the excess extract powder. The supernatant was carefully collected to be filled into the soft capsules. The AGPE-loaded SMEDDS was filled into a gelatin shell soft capsule (0.5 mL/cap) using an industrial pilot scale soft capsule filling machine at a pharmaceutical company, Kaewmungkorn Co., Ltd. (Ratchaburi, Thailand).

#### 2.2.7. Assay and Dissolution

The content of AGP was evaluated to determine the precise quantity of AGP in each capsule. The assay protocol was conducted following the *United States Pharmacopeia* (*USP43*) general chapter. In detail, the soft capsule shell was cut, and the sample solution was withdrawn and accurately weighed. Then, the solution was diluted in methanol, and the AGP content was quantified using HPLC. The % assay was calculated according to Equation (1) as follows:

% Assay = (actual quantified content of AGP × 100)/initial theoretical added content of AGP(1)

The dissolution test was conducted using a dissolution instrument (DT720, Erweka, Heusenstamm, Germany) with *USP* Apparatus 2 (paddle) in 900 mL of 2.5% sodium lauryl sulfate (SLS) and 0.1 N hydrochloric acid at 37 °C to simulate the physiological gastric environment. The paddle speed was set at 75 rpm. The dissolution medium was adjusted to contain SLS to allow AGP, which is a poorly water-soluble compound, to dissolve and provide a sink condition. At predetermined times of 5, 15, 30, 45, 60, 90, and 120 min, 5 mL of the release medium was sampled and replaced with fresh medium to maintain a constant volume. The tested solutions were then diluted in methanol, and the amount of AGP dissolved was analyzed with HPLC.

#### 2.2.8. Uniformity of Dosage Unit

The uniformity of dosage units was conducted according to the *USP43* general chapter, which states that weight variation should be performed for solution-filled soft capsules. Accordingly, the protocol was carefully followed. The capsules were randomly sampled (10 capsules) and individually weighed to record each gross weight. Then, each capsule was cut with clean scissors and the content was completely removed. The empty gelatin shell was washed with methanol and the occluded solvent was wiped off and left to evaporate for 30 min, whereas complete removal of the SMEDDS was ascertained visually through the obtained clear gelatin shell. The shells were individually weighed to obtain the net content. The drug substance content was calculated as the percent label claimed with the assay content for the calculation of acceptance value (AV) using Equation (2):

(2) $$\mathrm{AV}\;=\;|M\;-\;\bar{X}|+\mathrm{ks}$$

where M is the reference value, k is the acceptability constant (equals to 2.4 when *n* = 10), and s is the sample standard deviation.

#### 2.2.9. Stability Study

The stability of the AGPE-loaded SMEDDS soft capsule was evaluated following the accelerated study protocol from the International Conference on Harmonization (ICH) of technical requirements for registration of pharmaceuticals for human use Q1A (R2) guideline and the ASEAN guideline on the stability of drug products. A sufficient number of soft capsules were contained in an aluminum tightly zipped bag and stored at 40 ± 2 °C with 75 ± 5% relative humidity. The stability testing on the physical appearance, assay, and uniformity of dosage unit was conducted at 0, 3, and 6 m of storage. The appearance was visually evaluated while the other experiments were performed, following the mentioned procedures.

#### 2.2.10. Ex Vivo Membrane Permeation Study

The permeation of the AGPE-loaded SMEDDS was examined to compare the permeation flux of the prepared system to the *A. paniculata* extract suspension. The colon of a pig was acquired freshly from the local slaughterhouse on the day of sacrifice and dissected into a circular shape with an area of approximately 2.5 cm^2^. Vertical Franz diffusion cells (diffusion area 1.77 cm^2^, receptor volume 6 mL) were used and the membrane was cramped between the donor and acceptor compartment of each cell with the lumen of the colon facing upward. Phosphate buffer saline (PBS) pH 6.8 with 2.5% SLS was filled in the receptor chamber with a magnetic bar for stirring, and any air bubble that occurred was removed before the investigation. The temperature of the medium was maintained at 37 ± 0.5 °C. The AGPE-loaded SMEDDS soft capsule was cut and dispersed in 50 mL PBS pH 6.8 to form a microemulsion, then 1 mL of the self-formed microemulsion was placed in the donor compartment. At various determined times of 0.5, 1, 2, 3, 4, 6, 8, and 24 h, 0.5 mL of the medium was withdrawn and fresh medium was replaced to maintain sink condition. The sampled medium at each timepoint was subjected to HPLC to quantify the content of AGP permeated. The relationship between AGP permeation and time was plotted to determine the steady-state flux (J) from the slope of the linear regression found. Also, the amount of drug permeated after 24 h per area (Q_24_/A) was calculated. The permeability coefficient was calculated using Equation (3).

(3) $$K_{p}=\frac{J}{C_{d}}$$

where K_p_ is the permeability coefficient, J is the steady-state flux, and C_d_ is the initial amount of drug in the donor compartment.

#### 2.2.11. Statistical Analysis

All experiments were performed in triplicate (*n* = 3). Data were reported as mean ± standard deviation (S.D.) The statistical significance was established at a 95% confidence interval after the analysis using an F-test and independent *t*-test or ANOVA with the least significant post hoc test.

## 3. Results and Discussion

### 3.1. Andrographolide Solubility Study

The solubility of AGP in the different oils, surfactants, and co-surfactants was studied by shaking the *A. paniculata* extract powder in the solvents for 72 h. The solubility findings are shown in Table 2. Since AGP is insoluble in water due to its nonpolar hydrophobic structure limiting its numerous beneficial biological activities, the drug was dissolved in different oils to determine the appropriate edible oil to be used in the SMEDDS formulation. Oleic acid, which was the only pure unsaturated oil investigated, proved to be the most promising as it could extract and dissolve the highest amount of AGP from the extract powder. Oleic could act as an oil phase in the ME system that entraps the hydrophobic active agent. The roles of surfactant and co-surfactant in the SMEDDS system were to form a liquid film at the interface of the oil and water phase, avoiding coalescence and improving overall miscibility of oil in the aqueous environment. For showing the highest AGP solubility, oleic acid, Tween^®^ 80, and PEG 400 were acquired in the construction of a pseudo-ternary phase diagram.

### 3.2. Ternary Phase Diagram

**Figure 1:**
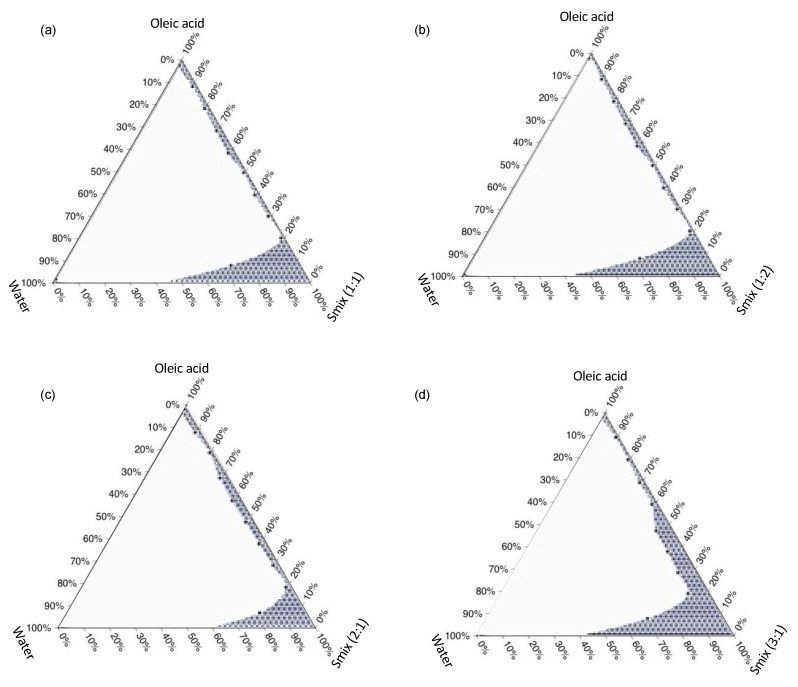
Ternary phase diagrams of oleic acid, water, and various S_mix_ (**a**) 1:1, (**b**) 1:2, (**c**) 2:1, and (**d**) 3:1. The ME areas are presented by the shaded regions.

The pseudo-ternary phase diagrams were constructed as preliminary information for an appropriate range of each mixture component that can be used to form MEs. The diagram helps to determine the range of oil, S_mix_, and water that can be mixed to formulate a stable ME. Accordingly, the ME can be created once the compositions are mixed at a proper ratio, as given by the diagram. The results are shown in Figure 1. The highest ME area was found with a surfactant; the co-surfactant ratio was 3:1. Upon using an S_mix_ of 1:1, the given ME area was a narrow range of water and oil content with high S_mix_ content (Figure 1a), whereas the area did not change when a higher amount of co-solvent was used (Figure 1b). Once a higher amount of Tween^®^ 80 was used (Figure 1c), the ME area was slightly larger for scantly higher oil content that could be used to form a stable emulsion. A satisfactory progression was shown when a greater surfactant amount was acquired. The S_mix_ of 3:1 presented a much larger ME area, where a wider range of water could be added once the oil phase was not greater than 50%, especially when reduced below 20%, as presented in Figure 1d.

These findings suggest that a stable ME or SMEDDS could be formed with an S_mix_ when greater Tween^®^ 80 ratios to PEG 400 are used. Together with S_mix_, the oil content should be less than 20% to allow for the formation of ME with a high water volume. This is a piece of essential information for developping an oral SMEDDS formulation where the oil and S_mix_ mixture would be added to the aqueous gastric content (usually 900 mL). Surfactants are one of the most important components in the ME preparations because they create a mechanical film and an electrical film (if charged) between the internal phase droplets (oil phase) and also reduce the surface tension [32,33]. Co-surfactants facilitate surfactants in creating a stronger barrier, decrease the rigidity of the surfactant interfacial film by reducing the bending stress of the interface, and give more flexibility to the interfacial film [34,35]. Accordingly, Tween^®^ 80 was the main component (with the aid of a long chain co-surfactant, PEG 400) for producing a physically stable system for showing no phase separation after centrifugation. Accordingly, Shah et al. (2018) acquired Tween^®^ 80 for the preparation of oral SMEDDS for nutraceutical delivery. The study proved that the system containing this non-ionic surfactant helped to increase the solubility of poorly water-soluble compounds and that >80% of the compound could be dispersed in 10 min [36]. These findings are useful for establishing oil, surfactant, and co-surfactant ranges for the optimization of SMEDDS.

### 3.3. Optimization of SMEDDS

From the data obtained from the pseudo-ternary phase diagram, the range between Tween^®^ 80, oleic acid, and PEG 400 was varied by an I-optimal design mixture type. The input range of surfactant (X_1_) was 50–80%, oil (X_2_) was 5–20%, and co-surfactant (X_3_) was 5–45%. The responses were droplet size (Y_1_), PDI (Y_2_), and zeta potential (Y_3_). A total of 16 runs were generated by DesignExpert^®^ software; each experimental run was conducted and characterized after dilution in high water content. It was found that all the SMEDDSs prepared were transparent, with no phase separation observed after the accelerated testing; the droplet size ranged from 15.4 to 147.6 nm, the PDI was narrow and ranged between 0.118 and 0.376, and the droplets presented a negative surface charge of oleic acid with zeta potential from −20.6 to −2.3 mV, as presented in Table 3. This suggested that the SMEDDS formulations created by DesignExpert^®^ software could be formed with various characteristics according to the compositions of each formulation. The data obtained were then acquired for the analysis of the effect of each component on SMEDDS for optimization.

**Figure 2:**
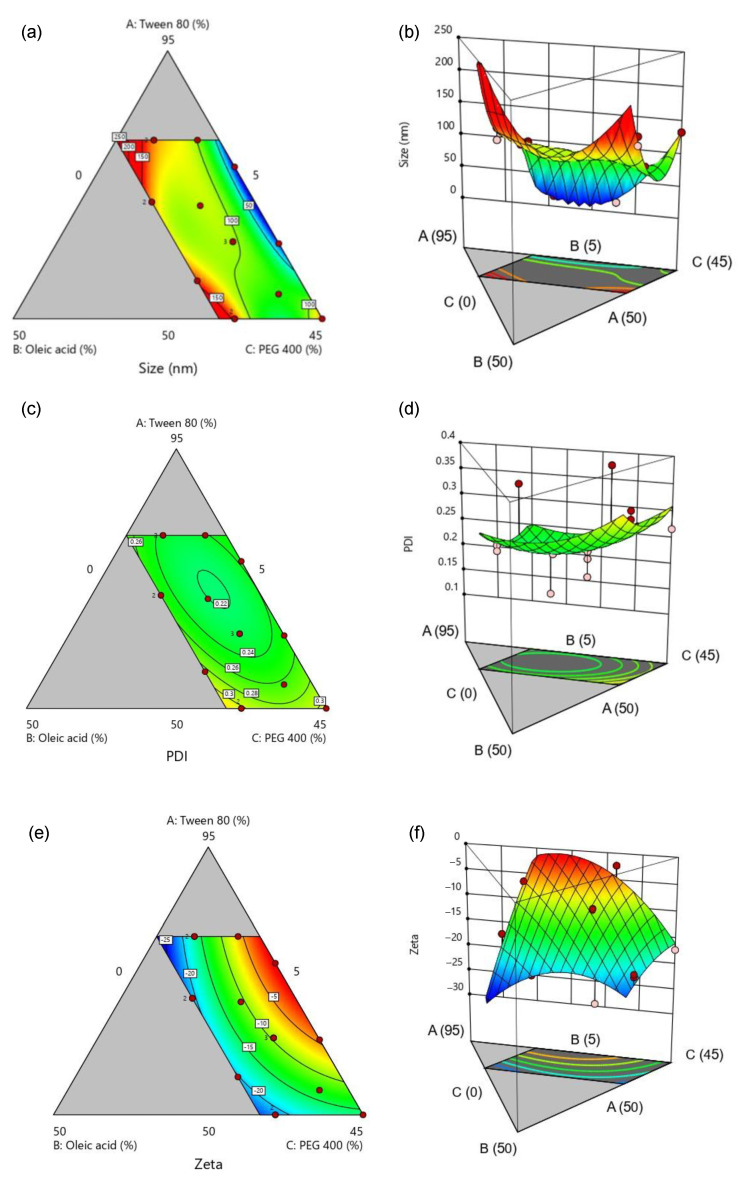
Two-dimensional contour plot and three-dimensional response surface area of (**a**,**b**) droplet size, (**c**,**d**) PDI, and (**e**,**f**) zeta potential of SMEDD composed of Tween^®^ 80, oleic acid, and PEG 400. The blue regions signify lower values, whereas, the gradient to green, yellow, and red indicate the higher values, respectively.

Considering the optimization of SMEDDS on droplet size, the 2D contour plot and the 3D surface plot (Figure 2a,b, respectively) showed that, once the percentage of oleic acid in the formulation was decreased, the droplet size would decrease if the ratio of Tween^®^ 80 to PEG 400 was approximately 3:1 (blue region). Despite low oleic acid content, the droplet size may increase when the ratio of surfactant and co-surfactant is closer to 1:1. This is in accordance with the S_mix_ ratio in the pseudo-ternary phase diagram. The PDI of the optimizing conditions was found to be insignificant with all areas in the plots (Figure 2c,d) around 0.2, which represented a rather homogeneous droplet size dispersion in any mixture [37]. Furthermore, the droplet zeta potential contour and surface plots (Figure 2e,f) depicted that the zeta potential moved towards a more negative value when a greater percentage of oleic acid was introduced in the formulation. This is reasonable, because the negative charge shown on the droplet surface was attributed to the carboxylic functional group in the fatty acid structure [38].

**Figure 3:**
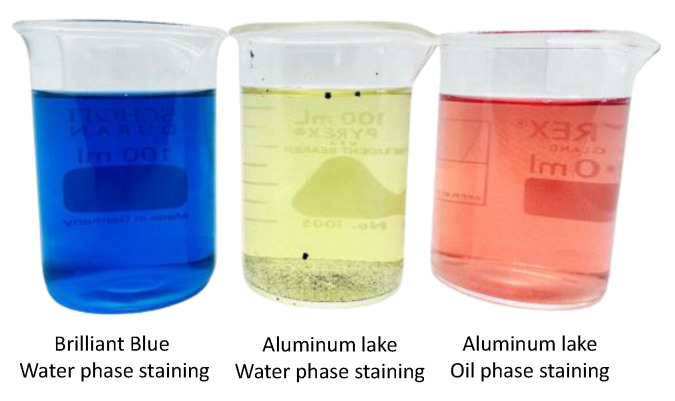
Confirmation of the type of ME formed through dyeing tests with water-soluble dye (brilliant blue) and water-insoluble dye (FD&C RED No. 40 aluminum lake).

According to the results of the 16 experimental runs from the DesignExpert^®^ program, the information was gathered and analyzed for the optimal condition for SMEDDS formulation. The criteria for obtaining the optimal composition are presented in Table 4. The SMEDDS components were set to be within the range of the optimization input. The droplet size was allowed to be within the range of the former results, as the majority of the size data were roughly 100 nm. The PDI was not considered in the optimization due to insignificant observation, while the zeta potential was within the negative range. These criteria were chosen as mentioned due to the characteristics of ME being nanosized stable droplets. The small and homogeneous droplet size would give a stable system, especially when considering sufficient surface charge to avoid coalescence. The optimized SMEDDS was composed of 68.998% Tween^®^ 80, with 13.257% oleic acid and 17.745% PEG 400. The desirability of this optimal condition from the selected criteria was ideal, showing excellent information quality [39]. Once the optimized condition of SMEDDS was conducted (*n* = 3), the actual result showed similarity to the predicted solution (*p* > 0.05). The droplet size was even less than <100 nm, which fit the definition of ME [30]. The zeta potential was negative, thus not showing coalescence or phase separation after centrifugation, referring to a physically stable system being developed. The centrifugation method has often been proven by the literature to ascertain the physical stability of the ME. Rathore et al., (2023) and Silberstein et al., (2023) conducted centrifugation to prove any instabilities of the SMEDDS [40,41]. This work performed the process at a harsher condition, thus no instabilities were found, ascertaining the strength of the Smix system. Interestingly, the optimized SMEDDS presented the ratio of surfactant and co-surfactant close to the S_mix_ of the ternary phase diagram (3:1). The ME system obtained from the optimized condition would yield an o/w ME system upon application in the gastric environment as there would be an excessive amount of aqueous medium compared with the oil phase. This was made certain through a dye test. It was found that the SMEDDS formed as O/W ME when visually inspected. After adding a water-soluble dye (brilliant blue), the color was dispersed throughout the ME and formed a clear blue dispersion. Once FD&C RED No. 40 aluminum lake (a water-insoluble color) was added, the color could not disperse and precipitated to the surrounding water during the continuous phase. On the other hand, a clear red dispersion was obtained when the aluminum lake was mixed with the SEMDDS formulation prior to the water being added. This confirmed that the aluminum lake stained the oil phase and dispersed homogeneously as a ME (Figure 3). Also, this could be determined by the type of surfactant and co-surfactant used (Tween^®^ 80 and PEG 400, respectively). Tween^®^ 80 is an o/w emulsifier, thus the o/w ME system could be formed. Hence, it was strongly confirmed in the preliminary finding and the optimization finding that the developed SMEDDS was at the optimal condition to be loaded with AGPE. Also, the optimized condition seemed to be promising, considering the safety of the formulation. Tween^®^ 80, PEG 400, and oleic have been and continue to be recognized as nontoxic and noncarcinogenic and are thus considered safe and well-tolerated regarding the intended amount to be taken [42,43,44]. Similarly, Huang et al. (2018) designed a SMEDDS formulation for nimodipinde delivery, using Tween^®^ 80 and PEG400 as surfactants. It was presented that SMEDDS can be optimized and is a promising strategy for poor water-soluble compounds causing low oral bioavailability [45,46]. This aligned with the purpose of our study, which focused on the delivery of AGPE.

Based on the solubility study, AGP was able to solubilize in Tween, PEG400, and oleic acid at concentrations of approximately 50.1, 54.8, and 1.6 mg/mL, respectively. The optimized SMEDDS was composed of 68.998% Tween^®^ 80, 13.257% oleic acid, and 17.745% PEG 400. In theory, it should be able to solubilize approximately 42 mg/mL of AGP, and we obtained a value of approximately 38 mg/mL from the preliminary study. Therefore, we set the target content of AGP in the SMEDDS as 30 mg/mL by calculating from the standardized amount of AGP in the extract (1 g of the dry extract contains 356 mg of AGP) added to the SMEDDS.

### 3.4. AGP Assay, Uniformity of Dosage Unit, Dissolution, and Stability Studies

The amount of AGP contained in the soft capsules was quantified following the pharmacopeial protocol. A mixture of oil and surfactants was prepared with a target AGP content of 30 mg/mL calculated from the content of AGP in the *A. paniculata* extract powder. Once 0.5 mL of the mixture was filled into the soft capsule, 15 mg of AGP then became the label amount of the prepared dosage form. It was found that the % assay of AGP in the soft capsules was 95.21 ± 1.70 %, which is preferable for the content of a drug in a plant-derived solid dosage form usually ranging from 90.0 to 110.0% according to the *United States Pharmacopeia 43* [47,48,49,50]. The amount of the formulation per unit was considered uniform after calculating the acceptance value. According to the *USP* guideline, the calculated AV was 5.68, which indicated that the uniformity of the dosage unit was compiled according to the guideline (acceptance criteria (L1) < 15.0, when *n* = 10). These results were gathered in a 0-month stability study and used to compare to 3-, 6-, and 12-month stability tests.

**Figure 4:**
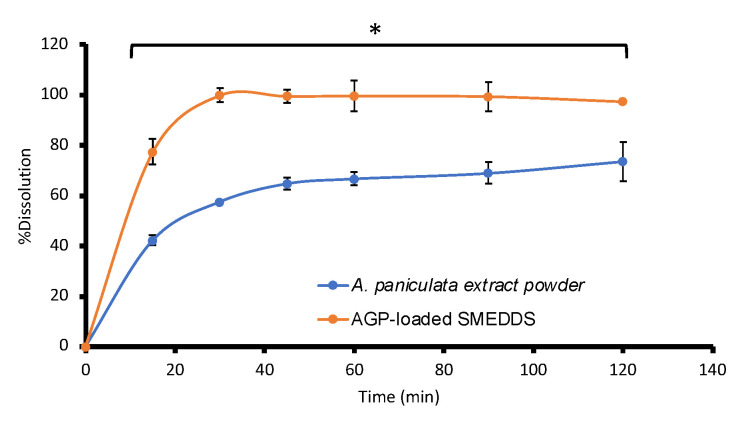
The dissolution profile of AGP from AGPE-loaded SMEDDS compared to *A. paniculata* extract powder-filled hard capsules (*n* = 6) in the gastric environment (pH 1.2) (* significant difference, *p* < 0.05).

The dissolution of AGPE-loaded SMEDDS capsules was studied and compared to *A. paniculata* extract powder-filled hard capsules with an equivalent amount of AGP. It was found that the SMEDDS preparation greatly enhanced the solubility of AGP (*p* < 0.05), where complete dissolution of the compound was reached at 30 min. Meanwhile, the solubilization of AGP in *A. paniculata* extract powder-filled hard capsules reached its limit after 45 min and only a maximum of 73 ± 7.78% was dissolved after 2 h (Figure 4), as the compound is very poorly soluble in an aqueous medium. The release condition used mimicked the gastric environment as it is the area where the formulation will first encounter and disperse to form ME [51]. Upon reaching the gastric fluid, microemulsion could spontaneously form at an interface between the oil droplets and the aqueous fluid because the required energy to form an emulsion is very low. The dissolution of AGPE-loaded SMEDDS also predicted that, upon the capsule intake, the ME could be self-formed in the gastric fluid after showing transparent dispersion of oil in the excess amount of liquid. During the process, AGP would expand in the aqueous medium as nanosized droplets of an oil, surfactant, and co-surfactant mixture; hence, the solubility of AGP increased [52]. The results are in accordance with Kim et al., (2019), who reported a significant increase in the solubility of methotrexate, which was completed within 20 min through SMEDDS formulation, where 53% of the drug powder dissolved after 60 min [53]. The developed SMEDDS has been a valuable strategy for improving the solubility of poorly water-soluble compounds, as described by several reports [54]. This work expands the knowledge regarding the aspects of herbal hydrophobic coumpounds and extracts. Also, the findings prove that the aqueous solubility of AGP could be improved using SMEDDS. This could be beneficial to patients/consumers, as a greater amount of the active compound can be dissolved and could lead to greater bioavailability. The production of SMEDDS could eliminate the rate-limiting step of drug absorption in terms of allowing the dissolution of AGP.

The stability of the prepared SMEDDS soft capsules was studied following an accelerated study protocol from the ASEAN guidelines on stability studies of drug products. The capsules were kept in a very warm and very humid environment controlled by an incubator. When visually observed, the physical stability of AGPE-loaded SMEDDS soft capsules was still intact, with unchanged color, odor, size, and shape. Furthermore, it was found that the formulation could still form a stable microemulsion in an acid medium by showing a clear solution after complete dissolution at all time points. The assayed content of AGP was similar to that before storage (97.83 ± 5.25% at 3 m, 103.30 ± 4.41% at 6 m, 98.42 ± 3.77% at 12 m, *p* > 0.05), suggesting that the AGP contained in the formulation was stable for at least 12 months. This allowed the prediction of the shelf-life of the formulation; however, larger production scale and process validation should be conducted to ascertain the stability. It should be noted that, as AGP is the primary component in the extract and is typically considered the active compound in *A. paniculata* formulations, our study focused on monitoring the AGP content to assess the stability of this crucial compound within the formulation. It is worth noting that we did not quantify other degraded products that might have resulted from the stress test. In fact, the content of related substances in the soft gel capsules after exposure to temperature and humidity stress should also be reported. The slightly higher content observed after storage could be attributed to a minor loss of vehicle content, possibly PEG, from the formulation into the capsule shell. Also, the uniformity of dosage analysis confirmed the homogeneity of the dosage form with an AV of 13.27, 12.22, and 11.67 for 3 m, 6 m, and 12 m, respectively, which is still within an acceptable limit. Although the AV value was in the acceptable range, the preparation of the SMEDDS and soft gelation capsule was performed on a pilot scale (large scale), in which higher variations than the lab scale could be observed. Thus, process modification and validation should be further conducted to control these parameters and provide lower AV values. The microemulsion could still be formed after the dispersion in an excess aqueous medium and undergo centrifugation. Therefore, the stability of the developed AGPE-loaded SMEDDS formulation was considerable and is promising for further studies.

### 3.5. Ex Vivo Membrane Permeation Study

**Figure 5:**
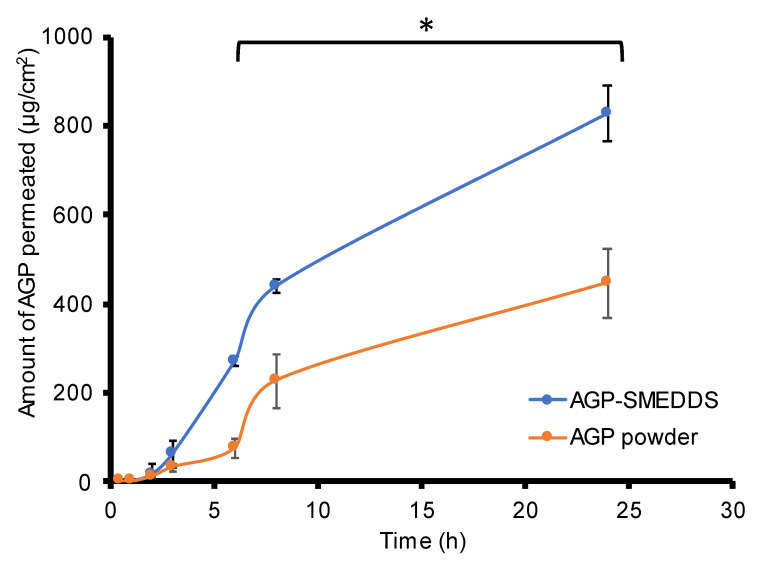
AGP intestinal absorption profile of AGPE-loaded SMEDDS compared to AGP from the *A. paniculata* extract (*n* = 4) (* significant difference, *p* < 0.05).

The membrane permeability of AGP was studied using a porcine intestinal membrane. Figure 5 and Table 5 illustrate the comparison of AGP permeation from the SMEDDS and *A. paniculata* extract powder. It was depicted that the permeation flux, permeability coefficient, and the quantity of AGP permeated at 24 h per area of the AGP from AGPE-loaded SMEDDS was twice as much as those findings from the AGP powder. This was owed to the increased solubility and surface area provided by the homogenous nanosized droplet dispersion of ME. SMEDDS is known to improve oral bioavailability of lipophilic drugs through different mechanisms such as increasing surface area, solubilization, altering membrane function by increasing membrane fluidity, or opening tight junction by surfactants and oils [55,56]. In our case, no report has yet mentioned the polysorbate effect on the alteration of membrane function [57]. Thus, it has been reported that the Tween^®^ 80 effect on increasing drug absorption was mainly due to increased solubilization of the drug and improved contact on the membrane surface, which possibly was herein the main mechanism of improved permeability [58]. Meanwhile, oleic acid has been reported to take part in improving the oral absorption and bioavailability of large molecule drugs as well as hydrophobic drugs, but the exact mechanism has not been concluded [59,60]. The proposed AGPE-loaded SMEDDS showed superior permeability compared with the control AGP for the component, and the developed system took part in improving the permeability of the drug through the intestinal membrane.

Several systems regarding *A. paniculata* and its extract have been investigated, including conventional dosage forms (tablets, capsules, syrups, tinctures, creams, pastes, aerosols, etc.) and advanced nanoformulations (e.g., liposome, polymeric nanoparticles, micelles). The advantages of the developed AGPE-loaded SMEDDS were to improve the solubility and intestinal membrane permeability of the active compound, AGP. The system overcame the drawbacks of conventional preparations concerning the low solubility that led to low oral bioavailability. Furthermore, it possessed benefits obtained through the use of a novel drug delivery system, such as improving the dissolution of the active ingredient and facilitating the permeation. Yet, the developed AGPE-loaded SMEDDS proved to be very promising for further clinical investigation for marketing purposes through scale-ups and intense examinations. Potential limitations for the system are to objectively prove that the formulation is effective and clinically safe and to uncover a suitable production process and parameters that can minimize variations in large-scale production.

## 4. Conclusions

The solubility of AGP in various solvents has been investigated, where oleic acid, Tween^®^ 80, and PEG 400 showed to be the most promising oil, surfactant, and co-surfactant for SMEDDS development, respectively. The ternary phase diagrams were constructed to picture the ME occurring area among the components. The SMEDDS formulation was optimized using an experimental design in which the optimal condition was acquired to be loaded with AGPE and filled into soft capsules. The AGPE-loaded SMEDDS proved to increase drug dissolution and membrane permeation whilst maintaining physical and chemical stability throughout the storage period under accelerated conditions. This study proposed the development and optimization of an AGPE-loaded SMEDDS formulation with the dissolution of a permeation enhancement. Also, ease of scale-up for industrial manufacturing was illustrated. The developed SMEDDS could be proven to increase drug absorption and bioavailability in further vivo studies. This development has been crucial to prove that the properties and delivery of a significant compound, AGP, from a natural source can be empowered by means of novel knowledge and technologies. The findings portray an optimized and auspicious formulation to be applied in clinical studies for safety and efficacy and to be further used to enrich the healthcare system.

## Figures and Tables

**Table 1:** List of oils, surfactants, and co-surfactants used.

Oil	Surfactant	Co-Surfactant
Coconut oil Maize oil Olive oil Soybean oil Sunflower oil Oleic acid	Cremophor RH40 Transcutol^®^ P Labrasol^®^ Tween^®^ 20 Tween^®^ 80	Ethanol PEG400 Propylene glycol Glycerin

**Table 2:** AGP solubility in different oils, surfactants, and co-surfactants (* significant difference, *p* < 0.05).

Oil		Surfactant		Co-Surfactant	
Solvent	Solubility (mg/mL)	Solvent	Solubility (mg/mL)	Solvent	Solubility (mg/mL)
Coconut oil	0.16 ± 0.02	Chromophore RH40	25.24 ± 2.96	Ethanol	26.33 ± 1.07
Corn oil	0.10 ± 0.01	Translutol^®^ P	27.08 ± 1.69	PEG 400	54.76 ± 4.92 *
Olive oil	0.22 ± 0.03	Labrasol^®^	24.73 ± 0.75	Propylene glycol	33.24 ± 3.16
Soybean oil	0.32 ± 0.02	Tween^®^ 20	29.20 ± 0.94	Glycerin	6.86 ± 1.41
Sunflower oil	0.16 ± 0.01	Tween^®^ 80	50.14 ± 4.23 *		
Oleic acid	1.64 ± 0.02 *				

**Table 3:** Experimental runs generated for SMEDDS optimization using DesignExpert^®^ software.

RUN	X_1_ Tween^®^ 80	X_2_ Oleic Acid	X_3_ PEG 400	Y_1_ Droplet Size (nm)	Y_2_ PDI	Y_3_ Zeta Potential (mV)
1	63	11.5	25.5	105.40	0.1770	−9.30
2	80	14.5	5.5	136.50	0.2310	−14.60
3	56.4	20	23.6	135.20	0.2730	−24.90
4	69.6	20	10.4	129.60	0.2710	−20.60
5	80	8	12	99.90	0.3360	−5.80
6	54.1	9.3	36.6	85.30	0.2760	−13.70
7	80	14.5	5.5	118.20	0.2200	−19.60
8	69	13.3	17.7	97.00	0.2200	−12.30
9	63	11.5	25.5	104.90	0.2260	−9.50
10	50	17.8	32.2	147.60	0.3240	−19.90
11	50	18	32	132.00	0.2650	−17.40
12	75.5	5	19.5	16.50	0.1180	−1.50
13	50	17.8	32.2	134.00	0.3080	−19.30
14	62.6	5	32.4	15.40	0.3760	−2.30
15	69.6	20	10.4	132.20	0.2520	−18.40
16	63	11.5	25.5	106.80	0.2120	−9.20

**Table 4:** Optimization criteria for SMEDDS formulation and the responses of the optimized formulation.

Factors	Goal	Lower Limit	Upper Limit	Solution	Desirability	Results	*p*-Value
Tween^®^ 80 (%wt)	is in range	50	80	68.998	1.000		
Oleic acid (%wt)	is in range	5	20	13.257			
PEG 400 (%wt)	is in range	0	45	17.745			
Size (nm)	is in range	80	115	101.68 ± 21.53		97.1 ± 0.76	0.75
PDI	none						
Zeta potential (mV)	is in range	−24.9	−10	−10.76 ± 2.38		−9.0 ± 0.95	0.30

**Table 5:** Intestinal membrane absorption parameters of AGPE-loaded SMEDDS compared to AGP from the *A. paniculata* extract (*n* = 4) (* significant difference, *p* < 0.05).

	J (µg·cm^−2^·h^−1^)	K_p_ (×10^−3^ cm^−2^·h^−1^)	Q_24_/A (µg·cm^−2^)
*A. paniculata* extract	19.90 ± 3.84	0.99 ± 0.19	447.34 ± 77.14
AGP-SMEDDS	35.49 ± 4.12 *	2.00 ± 0.23 *	827.87 ± 63.77 *

## Data Availability

The data presented in this study are available in this article.
